# Effectiveness of prolonged use of continuous passive motion (CPM) as an adjunct to physiotherapy following total knee arthroplasty: Design of a randomised controlled trial [ISRCTN85759656]

**DOI:** 10.1186/1471-2474-7-15

**Published:** 2006-02-23

**Authors:** Anton F Lenssen, Yvonne HF Crijns, Eddie MH Waltjé, George M Roox, Mike JA van Steyn, Ruud JT Geesink, Piet A van den Brandt, Rob A de Bie

**Affiliations:** 1 University Hospital Maastricht, Department of Physical Therapy, Maastricht, The Netherlands; 2 University Hospital Maastricht, Department of Physical therapy, Maastricht, The Netherlands; 3 University Hospital Maastricht, Department of Orthopaedics, Maastricht, The Netherlands; 4 Maastricht University, Department of Epidemiology, Maastricht, The Netherlands

## Abstract

**Background:**

Adequate and intensive rehabilitation is an important requirement for successful Total Knee Arthroplasty. The primary focus of early rehabilitation is ambulation of patients and regaining range of motion in the knee.

Although research suggests that Continuous Passive Motion should be implemented in the first rehabilitation phase following surgery, there is substantial debate about the duration of each session and the total period of CPM application and. A Cochrane review on this topic concluded that short-term use of CPM leads to greater short-term range of motion. It also suggested, however, that future research should concentrate on the treatment period during which CPM should be administered.

**Methods:**

In a randomised controlled trial we intend to investigate the efficacy of prolonged use of a continuous passive motion (CPM) device in the home situation as an adjunct to standardised physical therapy. The experimental treatment is compared to standardised physical therapy, in patients with osteoarthritis of the knee undergoing Total Knee Arthroplasty (TKA). Efficacy will be assessed in terms of faster improvements in range of motion and functional recovery.

Seventy patients with knee osteoarthritis undergoing TKA and experiencing early postoperative flexion impairment (less than 80° of knee flexion at the time of discharge) will be randomised over two treatment groups, a usual care group and an experimental group

The experimental group will receive CPM + physiotherapy for 17 consecutive days after surgery, whereas the usual care group will receive the same treatment during the in-hospital phase (i.e. about four days), followed by physical therapy alone (usual care) in the first two weeks after hospital discharge.

From 18 days to three months after discharge, both groups will receive standardised PT. The primary focus of rehabilitation will be functional recovery (e.g. ambulation) and regaining range of motion (ROM) in the knee.

**Discussion:**

Because restricted knee ROM affects functional activities, knee ROM and knee function are regarded as the primary indicators of successful TKA. Potential effects of the intervention under study include rapid return of knee flexion accompanied by earlier return to functional activities of daily life. If patients benefit significantly from prolonged CPM use, this treatment should be added to the standard PT treatment at home.

We expect the additional home CPM programme to be more effective than the usual physiotherapy programme, resulting in a difference in ROM of at least 5°, 17 days after surgery. This clinically important difference, with a possible flexion ROM of about 100°, is expected to lead to better functioning in activities of daily life, like walking, and earlier ability to cycle. These advantages should result in earlier and increasing independence.

## Background

With the ageing of the population, the prevalence of degenerative joint diseases is increasing. Reports show that over a one-year period, 25% of people over 55 years have a persistent episode of knee pain, of whom annually about one in six consult their general practitioner, in both the UK and the Netherlands. The prevalence of painful disabling knee osteoarthritis in people over 55 years is 10%, of whom one quarter are severely disabled. In all, over 300,000 Dutch residents currently suffer from knee osteoartritis (OA). Total Knee Arthroplasty (TKA) is a common intervention that can enhance the quality of life for patients with knee OA. Over 7500 TKAs are performed in Dutch hospitals every year. In 2003, 160 TKAs were performed at the Maastricht University Hospital.

Adequate and intensive rehabilitation is an important requirement for successful TKA. The primary focus of early rehabilitation is ambulation and regaining range of motion (ROM) in the knee. Because restricted knee ROM affects functional activities, knee ROM is regarded as one of the primary indicators of a successful TKA. Rapid return of knee flexion accompanied by earlier return to functional activities of daily life is one of the potential effects of the intervention we are proposing to study.

Although research findings favour the use of Continuous Passive Motion (CPM) in the first rehabilitation phase following surgery [1-4], there is substantial debate about the total period of CPM application and the duration of individual sessions. A Cochrane review [1] on the topic concluded that short-term use of CPM leads to a more rapid recovery of rabnge of motion. It also suggested, however, that future research should concentrate on the period during which CPM should be administered, and therefore called for research into longer-term use, involving long-term follow-up.

Before the year 2000, discharge from the Maastricht University Hospital after TKA was scheduled approximately 14 days after surgery. Nowadays, most patients are discharged four days after surgery. Since the time spent in hospital following surgery has decreased, continuation of CPM after hospital discharge might be beneficial. Although CPM is increasingly being administered in the postclinical home situation in recent years and is beginning to become part of the usual care programme, proper research into the effectiveness of a prolonged use of CPM at home is still lacking [1,2]. The only study that has been reported [5] compared CPM with physical therapy (PT) as a stand-alone therapy, whereas in the study presented here, CPM will be added to a standardised programme, adequately reflecting current practice.

This study is expected to provide new insights into the additional effects of CPM and to provide evidence to justify or refute its use.

Currently, orthopaedic surgeons at the hospital and physiotherapists at the hospital and at home play an important role in the rehabilitation process for TKA patients. The proposal presented here involves the same health care professionals and the same treatment strategies currently in use, but one patient group will additionally receive CPM at home.

We expect the effect of CPM treatment to be a quicker restoration of ROM, resulting in improved ADL function during the first 3 months after surgery.

Knee flexion values of 95° and 105° are regarded as range of motion benchmarks [6] in the functional recovery following CPM. While 95° of knee flexion allows normal ADL function, 105° of flexion gives a person the opportunity to ride a bicycle. This is of great advantage both in daily life (at least in the Netherlands) and in the rehabilitation from TKA surgery, because cycling allows patients to move the knee much more. We expect that prolonged use of CPM at home will allow patients to achieve these ROM benchmarks earlier in their recovery process.

About 50% of the patients undergoing one of the 160 TKAs performed annually at the Maastricht University Hospital have less than 80° of flexion 4 days after surgery and therefore potentially meet the inclusion criteria of the proposed study.

If the study should find that patients benefit from home CPM, this treatment should be added to the standard PT treatment currently administered. No differences in direct costs of rehabilitation are expected from implementation of the study results.

### Objective

Continuous passive motion (CPM) has proved to increase the amount of knee flexion for knee patients in the acute hospital setting (5–10 days). The primary purpose of the proposed randomised controlled trial is to establish whether there is additional long term benefit of continuing CPM after hospital discharge.

### Research question

What is the effect on range of motion and functional status of prolonged use of a continuous passive motion device at home in addition to physical therapy, compared to physical therapy alone, in patients with limited flexion range of motion (less than 80°) of the knee at discharge from the hospital following total knee arthroplasty?

## Methods/Design

### Study design

The proposed study is a randomised controlled trial, with blinded treatment allocation, assessment and analysis, in which 70 patients undergoing a scheduled unilateral TKA will be evaluated to assess the added value of CPM at home, using function and mobility as the main outcomes.

### Participants

We intend to include 70 patients in the study, who must be eligible for unilateral primary TKA as a result of osteoarthritis of the knee. Patients will be included if they have less than 80° of flexion range of motion 4 days after surgery, are able to understand and speak Dutch, are not suffering from mental disabilities and are resident inside the 'Maastricht heuvelland' region. Patients will be excluded if they need to stay in hospital for more than 5 days after surgery or show relevant co-morbidity influencing mobility (e.g. claudication, other prosthesis) or are operated upon using minimally invasive surgery.

Eligible patients will be contacted one week before planned surgery, and will be randomised into two groups after signing an informed consent form, on the day they are discharged from hospital. Patients will thus have 10 days in which to consider participation.

Blocked and concealed randomisation with a block size of 4 will ensure equal distribution of patients over the two treatment groups. Groups will be prestratified on preoperative flexion mobility of the knee.

### Interventions

During the in-hospital period, all patients will receive a standardised PT programme, involving 20 minutes of PT per day for four days. During the first two weeks after discharge, patients in the experimental group will receive CPM for 4 hours a day in addition to regular PT treatment. Patients in the control group will receive only regular PT treatment (usual care).

From day 18 onward, all patients will receive regular PT treatment until 3 months after surgery, if indicated.

The post-clinical PT will be standardised in terms of treatment objectives. All patients will receive treatment consisting of active and passive mobilisation of the knee joint, active strengthening of the m. quadriceps, and training of ADL functions (gait, sit to stand and stair climbing).

Outcome assessment

After baseline variables have been collected, outcome measures will be assessed at 17 days, 6 weeks and 3 months after surgery, during normal routine assessments at the orthopaedic clinic (table 1). The outcome assessor will be blinded.

Primary outcome measures are:

1. functional status, using the WOMAC function score [7,8] and the Knee Society Score[9]; and

2. range of motion, assessed with a long-arm goniometer [10].

Secondary outcome measures are:

a. perceived effect, using a 7-point Likert scale;

b. postoperative medication use (amount; type will be standardised);

c. satisfaction with treatment, on an 11-point Likert scale;

d. satisfaction with treatment result, on an 11-point Likert scale;

e. adherence to treatment protocols and use of CPM (in hours), measured with a patient diary;

f. quantity, duration and nature of PT intervention.

**Figure 1:**
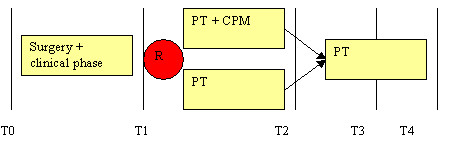
study design. Legend to figure 1: T0 one week before surgery. T1 4 days after surgery. T2 17 days after surgery. T3 6 weeks after surgery. T4 3 months after surgery.

The study design is depicted in figure 1

The first primary endpoint of the study is on day 17 after surgery, which is when the experimental treatment stops and short-term effects are measured.

The second endpoint is at 3 months after surgery.

Table 1 shows the timing of the outcome assessment

### Power analyses

We assume a difference of more than 5° of knee flexion mobility (SD 8°) at the end of the CPM application to be clinically relevant. With an alpha of 0.05, and a power of 80%, we will need 28 patients per group to prove this.

### Data analysis

Data will be stored and analysed with SPSS-12.0 After checks for missing values and for normality of the data, regression techniques will be applied by a blinded analyst using the 'intention-to-treat' principle. Primary and secondary outcome measures will be reported for the in-hospital and home situations, and for 6-week and 3-month follow-up.

The primary research question will be tested using Student's t-tests with a p-value of 0.05 being regarded as significant.

## Discussion

### Dissemination

The results of this trial are expected to provide evidence and clinically relevant information of direct use to professionals working in the field of rehabilitation after TKA (orthopaedic surgeons, general practitioners, physical therapists, occupational therapists and nurses). If the additional use of CPM proves efficacious, CPM should be administered to the target population. Therefore, health insurance companies and public health services should take note of the results and implement the findings in their activities.

The results of the research will be disseminated by means of publications in peer-reviewed scientific journals, by updating the Dutch arthrosis guidelines for GPs and physical therapists, by publication of a PhD thesis and by menas of lectures and presentations at conferences.

The relevant parties that can assure proper dissemination of the research findings are to a large extent represented in the project group. The orthopaedic surgeons are involved in drawing up the osteoarthritis guidelines, the research physical therapist and the project leader are responsible for the osteoarthritis guidelines for physical therapists, and the PhD student and promotion team are responsible for the scientific dissemination of the results. The patients with osteoarthritis receiving TKA surgery will receive updated information through an updated leaflet, made available at the hospital.

### Ethics

Patients will be asked to participate in the study one week prior to the planned surgery date. They will receive verbal and written information on the study. After doing so, they will have a 10-day period in which to decide whether or not to participate. Patients will be informed about the option to end their participation in the study at any time. An independent physician will be appointed to whom patients can turn if they have any questions, etc. Written consent will be asked prior to participation. All data will be collected by a research assistant and will be stored in a database. The data will be anonymised and coded so third parties will not be able to link data to specific patients, thus ensuring complete confidentiality of data of individual patients. After conclusion of the study, all participants will be informed of the outcome.

### Risks of participation

We believe there is very little additional risk for the participants included in the CPM treatment group. All patients will be familiar with the use of CPM device at the time they are discharged from the hospital. Nevertheless we have taken out supplementary insurance for all participants in the study.

## Competing interests

The author(s) declare that they have no competing interests.

## Authors' contributions

AFL has participated in the design of the study and will participate in the assessments and follow-ups, statistical analyses and writing. YC and EW have participated in design and will participate in the assessments and follow-ups. MvS, RG, PvdB and RdB have participated in the design. All authors have read and approved the final manuscript.

## Pre-publication history

The pre-publication history for this paper can be accessed here:



## Figures and Tables

**Table 1:** Timing of the outcome assessment

	T0 Pre-operative	T1 Clinical phase	T2 17 days after surgery	T3 6 weeks after surgery	T4 12 weeks after surgery
Range of motion	X	X	X	X	X
Knee Society Score	X	X	X	X	X
WOMAC	X		X	X	X
Perceived effect			X	X	X
Pain medication		X	X	X	X
Satisfaction with treatment			X	X	X
Satisfaction with results			X	X	X
Compliance			X		
Quantity, duration and kind of treatment			X	X	X
